# Mechanosensitive receptors in migraine: a systematic review

**DOI:** 10.1186/s10194-023-01710-1

**Published:** 2024-01-15

**Authors:** Adriana Della Pietra, Laura Gómez Dabó, Petr Mikulenka, Christian Espinoza-Vinces, Doga Vuralli, Isil Baytekin, Paolo Martelletti, Rashid Giniatullin, On behalf of the School of Advanced Studies of the European Headache Federation (EHF-SAS)

**Affiliations:** 1 A.I. Virtanen Institute for Molecular Sciences, University of Eastern Finland, Kuopio, Finland; 2 Neurology Department, Hospital Universitari Vall d’Hebron, Barcelona, Spain; 3 Department of Neurology, Third Faculty of Medicine, Charles University and University Hospital Kralovske Vinohrady, Prague, Czech Republic; 4 Department of Neurology, Clínica Universidad de Navarra, Pamplona, Spain; 5 Department of Neurology and Algology, Neuroscience and Neurotechnology Center of Excellence, Neuropsychiatry Center, Gazi University, Faculty of Medicine, Ankara, Turkey; 6 Department of Neurology, Bakirkoy Research and Training Hospital for Psychiatry, Neurology and Neurosurgery, Istanbul, Turkey; 7 School of Health Sciences, Unitelma Sapienza University of Rome, Rome, Italy

**Keywords:** Mechanotransduction, Migraine, Headache, Mechano-neurobiology, Piezo, K2P, ASICs, NMDA, TRP

## Abstract

**Background:**

Migraine is a debilitating neurological disorder with pain profile, suggesting exaggerated mechanosensation. Mechanosensitive receptors of different families, which specifically respond to various mechanical stimuli, have gathered increasing attention due to their potential role in migraine related nociception. Understanding these mechanisms is of principal importance for improved therapeutic strategies. This systematic review comprehensively examines the involvement of mechanosensitive mechanisms in migraine pain pathways.

**Methods:**

A systematic search across the Cochrane Library, Scopus, Web of Science, and Medline was conducted on 8th August 2023 for the period from 2000 to 2023, according to PRISMA guidelines. The review was constructed following a meticulous evaluation by two authors who independently applied rigorous inclusion criteria and quality assessments to the selected studies, upon which all authors collectively wrote the review.

**Results:**

We identified 36 relevant studies with our analysis. Additionally, 3 more studies were selected by literature search. The 39 papers included in this systematic review cover the role of the putative mechanosensitive Piezo and K2P, as well as ASICs, NMDA, and TRP family of channels in the migraine pain cascade. The outcome of the available knowledge, including mainly preclinical animal models of migraine and few clinical studies, underscores the intricate relationship between mechanosensitive receptors and migraine pain symptoms. The review presents the mechanisms of activation of mechanosensitive receptors that may be involved in the generation of nociceptive signals and migraine associated clinical symptoms. The gender differences of targeting these receptors as potential therapeutic interventions are also acknowledged as well as the challenges related to respective drug development.

**Conclusions:**

Overall, this analysis identified key molecular players and uncovered significant gaps in our understanding of mechanotransduction in migraine. This review offers a foundation for filling these gaps and suggests novel therapeutic options for migraine treatments based on achievements in the emerging field of mechano-neurobiology.

## Introduction

The origin of migraine pain is still highly debated, and its underlying mechanisms are not completely known. On one side, the migraine pain is believed to start in the peripheral trigeminovascular system while other studies suggest its central mechanisms [1–4]. In this complex system, the functional interactions, including chemical signaling and mechanical forces, between trigeminal ganglia (TG) neurons and glial cells, meningeal immune cells, pial/dural fibroblasts, and local vessels are enhanced during a migraine attack [5, 6]. The specific mechanical forces from the shear stress in dilated vessels and the regular vessel pulsations may be responsible for the mechanosensitive release of endothelial ATP [7] and, potentially, also of the key migraine messenger neuropeptide calcitonin gene related peptide (CGRP) from the perivascular nerves [8]. As shown in preclinical rodent models, both ATP and CGRP can directly induce mast cell degranulation, a process that can also be additionally activated by mechanical forces from blood pulsations [9–11]. Activated mast cells subsequently release a medley of pro-nociceptive compounds, including serotonin, histamine, cytokines, leukotrienes, prostaglandins, ATP, and nitric oxide, intensifying stimulation of nociceptive fibers and further amplifying CGRP release [12–15]. This interplay of chemical and mechanical forces can initiate a relentless vicious circle of neuronal sensitization and sterile inflammation, which supports the persistence of migraine pain [16].

In the central nervous system (CNS), cortical spreading depression (CSD) can also activate meningeal mechanoreceptors, contributing to the headache phase in migraine with aura [17]. This phenomenon is likely a consequence of the known association of CSD with oedema and brain swelling [18]. Additionally, CSD can impact the glymphatic (perivascular) outflow, responsible for clearing waste material from the brain and potentially inducing additional cortical swelling [19]. Apart from such direct activation of mechanoreceptors, CSD can play an indirect role in mechanotransduction supporting meningeal neurogenic inflammation by triggering the release of CGRP [20, 21] and substance P, activating mast cells [22, 23]. The generated sterile neuroinflammation can sensitize dural local nerves to mechanical stimuli [24]. On one hand, this hypersensitive peripheral state triggers orthodromic nociceptive signaling directed to the brainstem [23, 25]. These repetitive stimuli can also cause central sensitization leading to allodynia [26]. On the other hand, an antidromic electrical firing travelling back to the peripheral meninges could degranulate mast cells and trigger meningeal release of CGRP from trigeminal nociceptors [27] supporting inflammation and neuronal sensitization.

Clinically, patients suffering from migraine consistently report mechanical hyperalgesia, mechanical allodynia along with pulsating type of pain as the most disturbing symptoms [28]. Similar symptoms reflecting enhanced mechanical sensitivity can be revealed in animal models of migraine [29].

Furthermore, it is well known that migraine is predominantly affecting women, who often endure more severe and protracted attacks, resulting in extended recovery periods [30]. Therefore, given the disparities between genders in their prevalence of migraine mechanosensitivity, it is essential to delve deeper into the underlying factors that contribute to variations in attack frequency, intensity, and incidence. Consequently, the development of gender-specific preventive strategies and treatments addressing mechanical hyperalgesia and allodynia, becomes a pressing imperative.

Surprisingly, despite the obvious importance of mechanosensitivity in the pathophysiology of migraine and the extensive knowledge of the molecular mechanisms of eukaryotic mechanosensitive channels [31], few of these mechanotransducers have been studied in migraine as triggers of nociception. Thus, this review focuses on the role of mechanosensitive receptors in the mechanisms of pro-nociceptive peripheral sensitization in migraine, which has been studied much more than the complex central sensitization.

Overall, this systematic review aims to start filling this gap in our knowledge, by analysing the mechanosensitive mechanisms that have been explored in migraine studies to date.

### The spectrum of mechanosensitive receptors implicated in nociception

**Fig. 1:**
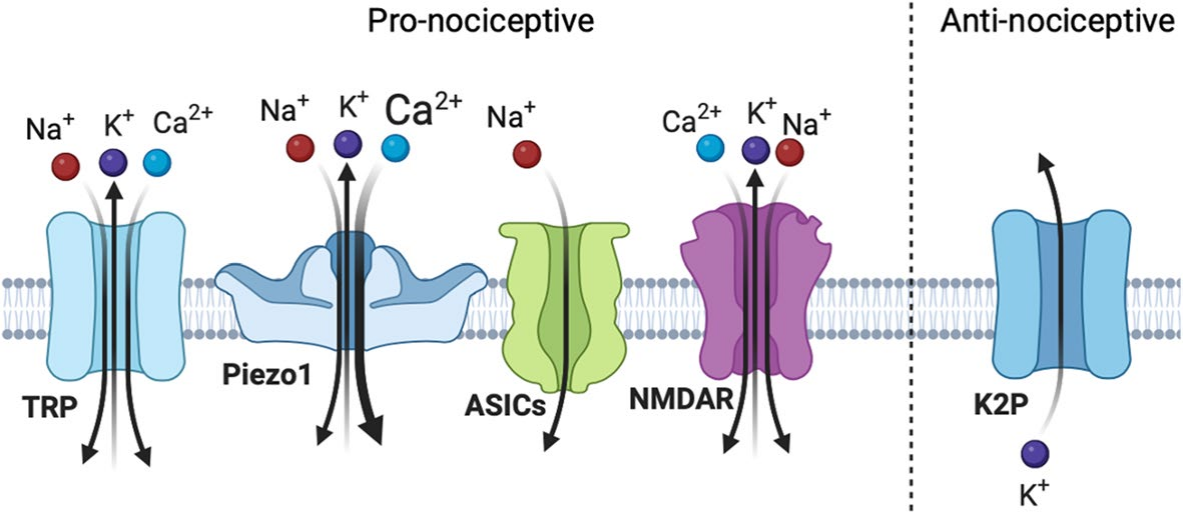
Mammalian mechanosensitive channels potentially implicated in nociception. The mechanosensitive channels could be divided into the pro-nociceptive TRP channels, Piezo1, ASICs and NMDA receptors, and the anti-nociceptive K2P channels

Figure 1 shows the studied mechanosensitive channels potentially implicated in pain pathways.

Among them, the members of the transient receptor potential (TRP) superfamily are the most studied mechanosensitive receptors in migraine, including transient receptor potential ankyrin 1 (TRPA1), transient receptor potential vanilloid-type 4 (TRPV4) and transient receptor potential canonical (TRPC) that contribute to mechanical hypersensitivity and are considered as possible therapeutic targets for migraine pain [32]. Various subtypes of TRP channels contribute to sensory transduction, thermosensation, taste, smell, vision, hearing, pain, and touch. TRP channels are also largely expressed on meningeal nociceptors and may respond to various exogenous and endogenous stimuli [33]. Thus, trigeminal sensory nerve fibers that innervate meninges, express TRPA1, TRPV1, TRPV4 and transient receptor potential melastatin 8 (TRPM8) channels [34, 35]. Activation of calcium permeable TRP channels effectively triggers the release of the pro-nociceptive CGRP from trigeminal nerve fibers [36]. However, these channels might not be ideal targets for drug development in migraine, considering that they are also necessary for normal tactile sensation, proprioception, and acute protective pain.

Instead of pro-nociceptive role of TRP channels, the two-pore-domain potassium channels (K2P) play rather the anti-nociceptive role. K2P channels are the principal governing factor of the background potassium conductance in the nervous system [37]. These potassium channels form ‘leak’ currents and are essential for the resting neuron membrane potential and regulating neuronal excitability. These channels appear to function to set the negative resting potential of TG neurons [38]. The heightened sensitivity to mechanical stimulation observed in TREK1 KO animals implies that these K2P channels play a crucial anti-nociceptive role in counterbalancing the inward currents generated by TRPV1 channels with which they are co-expressed [39]. Up to this point there have been 15 mammalian K2P potassium channels discovered. These channels are subdivided into 6 families as weak rectifying (TWIK), TWIK-related (TREK), TWIK-related acid-sensitive (TASK), TWIK-related alkaline pH-activated (TALK) and TWIK-related spinal cord potassium channels (TRESK) [40, 41]. Expression of these potassium channels has been discovered in nociceptive dorsal root ganglion (DRG) and TG neurons [38].

Piezo1/2 channels are mechanically sensitive gigantic non-selective cationic ion channels, which are highly calcium permeable [42]. Our knowledge on functions of these recently discovered Piezo channels is actively updating [43]. Interestingly, Piezo1 was involved in mediating the reduction of pain threshold caused by sleep deprivation, while microinjection of the Piezo1 antagonist GSMTx4 partially reversed the pain threshold [44]. In contrast, the other study showed that enhanced expression of Piezo1 channels in sensory neurons would reduce rather than cause mechanical pain responses [45]. However, only few studies connect these professional mechanotransductors with migraine, as shown in next sections of this review.

Additional candidates that will be addressed in this review are members of the mechanosensory abnormal/degenerin channel family, including acid-sensing ion channels (ASICs), which respond to both mechanical and acidic stimuli by opening sodium-permeable pores [46]. ASICs, apart from protons mechanical triggers, are activated by a variety of mediators e.g. cations, neuropeptides, arachidonic acid, protein kinases, and proteases [46]. They are expressed both in the brain and the peripheral nervous system [46]. However, conclusive evidence on whether ASIC activity is modulated directly by mechanical force is lacking.

N-methyl-D-aspartate (NMDA) receptors, which are linked to CSD mechanisms, can be activated by amphipathic molecules such as arachidonic acid (AA) but also by membrane stretch [47] suggesting them also as the mechanotransducers.

## Methods

### Study identification

This systematic review followed the PRISMA guidelines [48]. We performed our search on four electronic databases (the Cochrane Library, Scopus, Web of Science, Medline) on 8th August 2023. The search was carried out by an information specialist skilled in systematic reviews in the University of Eastern Finland. The following search string was used:

- #1 "Migraine Disorders"[mh]
- #2 migrain*[tw]
- #3 #1 OR #2
- #4 Biophysics[mh] OR "Biomechanical Phenomena"[mh]
- #5 biophysic*[tw] OR biomechanic*[tw] OR mechanobiolog*[tw] OR mechanosensitiv*[tw] OR mechanotransduct*[tw] OR "mechanical force*"[tw] OR "mechanical propert*"[tw] OR "mechanical stress*"[tw] OR "mechanical tension*"[tw] OR "physical force*"[tw].
- #6 piezo1[tw] OR "piezo 1"[tw] OR piezo2[tw] OR "piezo 2"[tw] OR trpm3[tw] OR "trpm 3"[tw] OR trpv4[tw] OR "trpv 4"[tw] OR trpc[tw] OR trek1[tw] OR "trek 1"[tw] OR traak[tw]
- #7 #4 OR #5 OR #6
- #8 #3 AND #7
- #9 #8 AND 2000:2023[dp] AND english[la]

### Study selection

The authors ADP and PM conducted individual assessments of all articles based on their titles and abstracts. Articles that could potentially meet the eligibility criteria in Table 1 of connecting mechanosensitivity and migraine pain passed the selection.

**Table 1:** Papers eligibility criteria

Inclusion criteria	• All kind of studies (cells, tissues, animal models) • Only full-text articles • Only original data articles • Publication dates: 2000–2023 • Involvement of mechanosensitive channels in migraine
Exclusion criteria	• Non-English articles • Reviews

**Fig. 2:**
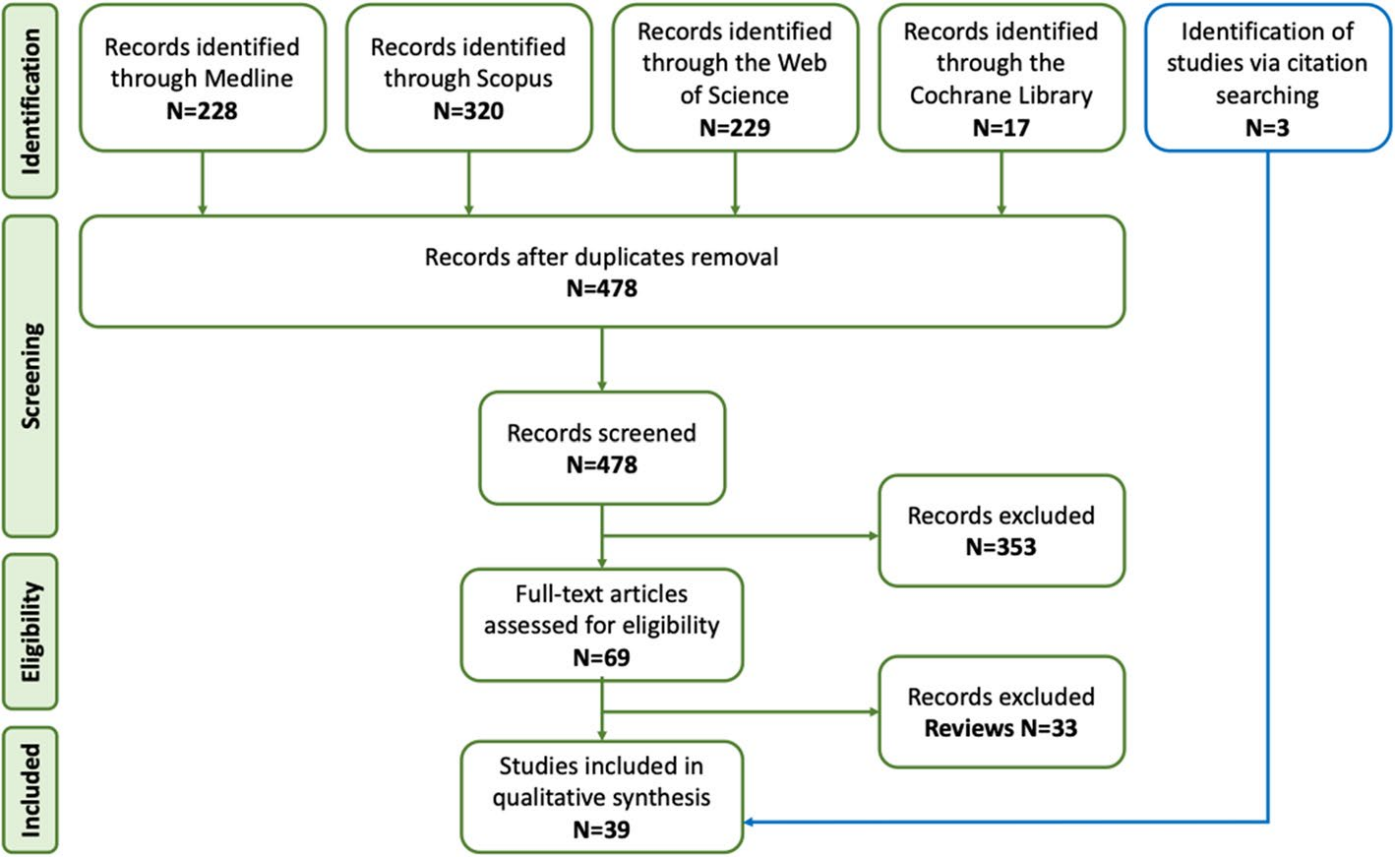
Article selection process

In cases in which disparities arose between the two assessors, those were resolved through discussions. Following this, a manual review of references of pertinent primary articles was carried out to identify any potentially additional eligible studies that might have been overlooked by the initial search strategy (Fig. 2).

### Emerging role of mechanosensitive receptors in migraine

It is now clear that mechanosensitive channels, widely present in our body, regulate many vital functions, including tactile sensations, proprioception and acute protective pain response [31]. Indeed, the extensive studies on polymodal (including mechanotransduction) TRP and highly mechanosensitive Piezo receptors led to a Nobel Prize in 2021 [49]. Notably, key migraine researchers won the prestigious Brain Prize that same year, reflecting recognition and growing interest in both areas of biomedical research. However, results of the current review indicate that, despite the wealth of knowledge in these apparently distinct domains, studies bridging the gap between mechanosensitive receptors and migraine remain relatively scarce.

Our search of reliable sources automatically selected 478 papers and reviews, which were reduced to 69 after selection based on titles and abstracts specifically focusing *on mechanosensitive receptors in migraine*. After excluding the review articles (33/69) and carrying out an additional screening of the full texts, the final number of original articles was 36. Additional 3 papers were included in the review after accurate citation searching, for a total of 39 articles.

**Fig. 3:**
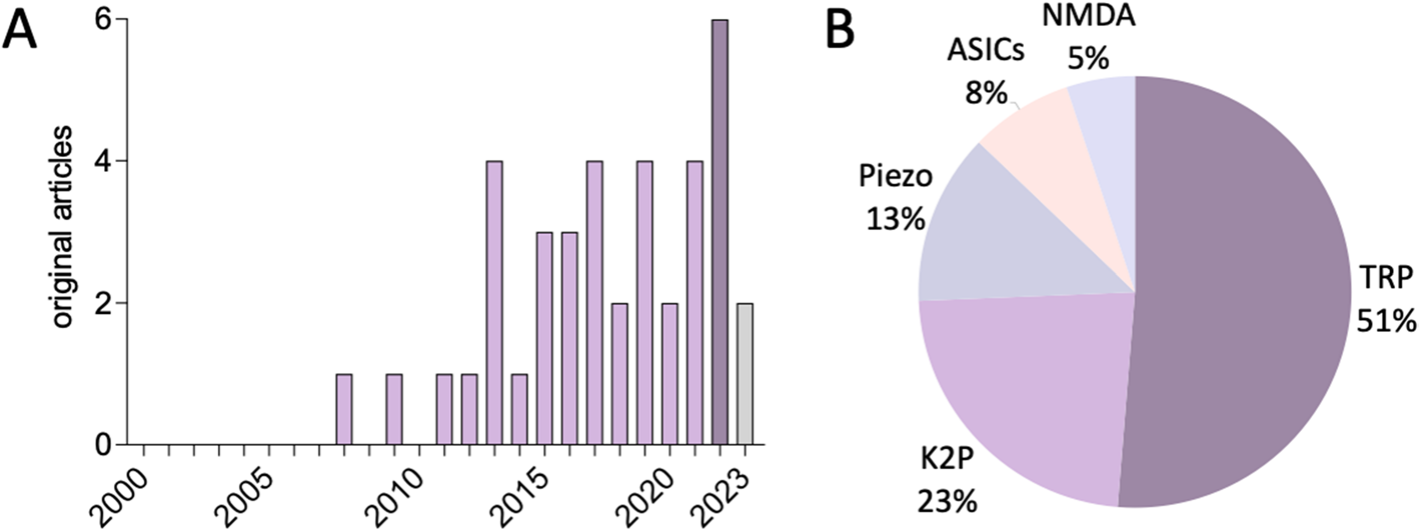
Statistics on the selected articles on mechanosensitive channels and migraine. **A** Publishing timeline of papers on mechanosensitive receptors in migraine. **B** Relative number of papers on the main mechanosensors involved in migraine pain

As shown in Fig. 3A, our analysis revealed an increasing research interest in mechanosensitive mechanisms involvement in migraine over time. One of first articles on TRP mechanosensitive receptors in migraine according to our search criteria, was published in 2007 [50]. The number of these articles raised especially after 2011, reaching peaks in 2013, 2017, 2019 and 2021 with 4 papers, and in 2022 with 6 original papers. In the current year 2023, still ongoing, only 2 papers have been published so far.

The resulting 39 original articles investigated the direct involvement of the specific type of mechanosensitive receptors in migraine mechanosensitivity and mechanical allodynia (Fig. 3B). Five mechanosensitive channels related to migraine pain have become the center of attention in recent times (Fig. 2B). Figure 3B shows that transient receptor potential (TRP) channels are the most studied group (20 papers), followed by two-pore-domain potassium channels (K2P, 9 papers), Piezo (5 papers), acid sensitive ion channels (ASICs, 3 papers), and N-methyl-D-aspartate (NMDA) receptors (2 papers).

### TRP and their modulators in migraine mechanosensitivity

In our selection, 20 papers link TRP channels to migraine, of which 17 were directly selected from the string and 3 more were retrieved from further the reference search.

Notably, TRPV1 is the best studied ion channel in nociceptors known to be activated by capsaicin [51], but also sensitive to other triggers such as (endo)vanilloids, acid and heat [36, 50] (Table 2). These stimuli can activate but also desensitize specific sensory nerve fibers, including those responsible for mechanosensitivity, releasing inflammatory neuropeptides [52]. TRPV1 is playing a significant role in sensory afferents in the stomach, intestine, and colon [53] and mediates nociception, pain hypersensitivity, and mechanosensitivity.

**Table 2:** Tested compounds promoting or counteracting migraine mechanical hypersensitivity and/or allodynia

TRP channels	Migraine mechanical hypersensitivity and/or allodynia		References
	**Activated by**	**Blocked by**	
TRPV1	Capsaicin Other vanilloids Acid Heat	Capsazepine JNJ-38893777 JNJ-17203212 XPro1595 SB-705498	[50, 54–56]
TRPV4	4α-PDD	RN1734 GSK-2193874	[57, 58]
TRPA1	Mustard oil Umbellulone Cinnamaldehyde	HC-030031	[58, 59]
TRPM3	Pregnenolone sulfate CIM0216	progesterone estrogenes	[59]
TRPM8	Cold Cooling substances (e.g. menthol)	AMG2850 RGM8-51 AMTB	[58, 60–62]
TRPC4	Lysophosphatidylcholine	ML204	[63]
TRPC5		CFA injections Paclitaxel	[56, 64]

Importantly, TRPV1 receptors is clearly implicated in modulation of mechanical pain in migraine [65]. TRPV1 was found to be abundant in the arterial walls of individuals suffering from chronic migraines [66]. The increased presence of TRPV1 receptors promoted the sensitivity of arteries to painful stimuli [66]. TRPV1 receptors were tested in animal migraine models treated with inflammatory soup [54], which promoted sensitization of the trigeminal nociceptive system. This sensitization has been associated with the development of headache and was likely the underlying mechanism of allodynia [24, 67]. In this study, the TRPV1 antagonists JNJ-38893777 and JNJ-17203212 (Table 2) efficiently inhibited trigeminal activation [54]. However, the failure in clinical trials, of the TRPV1 antagonist SB-705498 to reduce capsaicin-evoked hyperalgesia [55] suggests that TRPV1 activation alone may not be the sole trigger of migraine.

Consistent with this, both in the WT and in TRPV1 knockout mice, modelling of migraine with the repetitive nitroglycerin (NTG) injections produced mechanical allodynia in the hindpaw but not in the face along with facial but not hind paw cold allodynia [68]. Therefore, the authors concluded that different peripheral hypersensitivities develop in the face versus hindpaw in this model [68].

In a Complete Freund’s Adjuvant (CFA) orofacial pain model in mice, it has been shown an increase in TRPV1 mRNA and protein immunoreactivity in TG neurons [56]. The study also found that the selective anti-soluble tumour necrosis factor alpha (TNF-alpha) compound XPro1595, reduced CFA-induced mechanical hypersensitivity in the orofacial region [56] suggesting the role of TNF-alpha in enhanced trigeminal mechanotransduction.

Another member of the TRP family, the TRPV4 channel responds to mechanical stimuli and changes in osmolarity [57]. Activation of TRPV4 channels in the rat dura has been shown to cause pain-like behavior, cephalic and extracephalic allodynia reflecting aberrant mechanical sensitivity, which was blocked by the TRPV4 antagonist RN1734 (Table 2) [57]. TRPV4 function in sensory neurons could be modulated downstream of the protease-activated receptor 2 (PAR2) signalling [69]. Consistent with the role in migraine mechanotransduction, PAR2 activation sensitizes meningeal nociceptors to mechanical stimulation [70]. Moreover, it has been found that PAR2 induced headache behaviours in mice was blocked by the selective PAR2 antagonist and was absent in PAR2 knockout mice [71]. Given the link between TRPV4 channels and PAR2 signalling, these studies provide indirect evidence that TRPV4 activity on meningeal nociceptors may contribute to headache, but it remains unclear whether TRPV4 plays a direct role in the ability of meningeal afferents to detect pressure changes. Thus, more studies are needed to better explore the potential role for this channel in migraine.

A polymodal TRPA1 channel in sensory neurons appears to be involved in pain transduction [59] since 42% and 38% of the rat dural afferents reacted to TRPA1 agonists mustard oil (MO) and umbellulone (UMO, Table 2) [59]. Application of 10% MO and 10% UMO to meninges resulted in a significant facial and hindpaw mechanical allodynia, sensitive to the TRPA1 antagonist HC-030031 (Table 2), which prevented cutaneous allodynia [59]. MO and UMO application to dura caused decreased exploratory rearing behavior, which was also sensitive HC-030031 [59]. These data suggested a possible role of TRPA1 channels in migraine mechanical pain. Moreover, TRPA1-mediated activity is likely involved both in migraine aura related phenomenon of CSD and sensitization of trigeminovascular system [72]. Interestingly, a focused study in rat and Rhesus monkey, of the selected fraction of meningeal afferents called ‘non-arterial diffuse dural innervation’ did not reveal immunolabeling of TRPV1 and TRPA1 receptors [73] suggesting their specific distribution in the meninges.

Transient receptor potential melastatin 3 (TRPM3) channels are widely expressed in human sensory neurons [74]. Notably, this mechanosensitive channel could be blocked by the female sex hormones oestradiol and progesterone [75]. Consistent with the role in migraine, two different selective TRPM3 agonists activated nociceptive firing in trigeminal nerve fibers in meninges [76]. Notably, however, that the nociceptive firing induced by TRPM3 agonists pregnenolone sulfate (PregS) or CIM0216 (Table 2) was much more prominent in female mice than in males [76]. This was in sharp contrast to the sex-independent activation of Piezo1 or TRPV1 channels in meningeal afferents. Advanced cluster analysis of meningeal spikes showed a sustained activation of nerve terminals mediated by TRPM3 channels with large-amplitude spikes specific in female mice, proposing a specific mechanosensitive profile in females. These findings suggest that TRPM3 channels may be involved in the generation of migraine pain, particularly in females [76].

Cold sensitive TRPM8 receptors, localized in small-diameter sensory neurons [60], are activated apart from cool temperatures, also by cooling substances such as icilin and menthol (Table 2) [60]. These channels appear to play a role in enhancing the transmission of mechanical sensory signals through C-fibers in the urinary bladder [77]. Moreover, it is known that many cold sensitive neurons also exhibit mechanosensitivity [78]. However, one study found that mice with ablation of TRPM8 neurons did not exhibit impairments in immediate mechanical responses [79]. The other investigation showed that TRPM8 channels activated by icilin evoke cutaneous allodynia [80]. In a study utilizing fluorescent tracer Fluoro-Gold within TRPM8^EGFPf/+^ mice to label dural afferent neurons, where migraine headache originates, Ren et al. (2018) surprisingly reported that only 3–4% of dural afferent neurons expressed TRPM8 channels. Therefore, while a significant proportion of dural afferent neurons do exhibit mechanosensitivity [81], TRPM8 expressing neurons are likely not represent an essential fraction of meningeal afferents [82]. AMG2850, a TRPM8 antagonist (Table 2), did not reverse CFA induced mechanical hypersensitivity or sciatic nerve ligation induced allodynia in rats [61]. These observations diminish the potential of TRPM8 antagonism as a promising therapeutic approach for migraine management. However, β-lactam derivative with TRPM8 antagonist activity, RGM8-51 (Table 2), decreased menthol induced neuronal firing in a primary culture of rat DRG neurons and mitigated, in a sex-dependent manner, the NTG-induced mechanical hypersensitivity in a in vivo NTG mouse model of chronic migraine [62].

TRPC4 channel is expressed in primary sensory neurons and associated with itching and pain [83]. A specific TRPC4 antagonist, ML204 decreased mechanical hypersensitivity in NTG acute and chronic migraine models in male and female mice and reduced migraine-like pain behaviours in both male and female mice in chronic NTG migraine model [63].

TRPC5 is another TRP channel from the same subfamily, expressed in sensory neurons that has been shown to mediate mechanical sensitivity and spontaneous pain in mice [64]. Table 2 lists lysophosphatidylcholine (LPC) as an endogenous agonist of both human and mouse TRPC5 [64]. In this study, it was found LPC appears to be an endogenous mediator of TRPC5 induced mechanical allodynia. It has been also shown that this compound was elevated in skin two hours after intraplantar injections of CFA and injury site-specific elevation in LPC were also shown in hindpaw of mice after incision [64]. Finally, it has been shown that TRPC5 associated mechanical allodynia was initiated after site specific increase in LPC [64].

Several papers simultaneously considered several types of above mentioned TRP channels in migraine related CGRP release. Thus, it has been revealed that capsaicin, TRPV1 agonist, cinnamaldehyde, TRPA1 agonist, TRPM8 agonist menthol could induce CGRP release from meningeal trigeminal afferents, TG and trigeminal nucleus caudalis (TNC) [58]. In the same study, mechanosensitive TRPV4 channels agonist 4α-PDD (Table 2) was also shown to induce a significant CGRP release from dural trigeminal afferents and TNC [58]. Moreover, the TRPV1 antagonist capsazepine, TRPA1 antagonist HC-030031 and TRPM8 antagonist AMTB (Table 2) blocked CGRP release from both peripheral (dura and TG) and central (TNC) parts of the trigeminovascular system implicated in generation of migraine pain. Likewise, the TRPV4 antagonist GSK-2193874 (Table 2) inhibited the release of CGRP from meningeal trigeminal nerve afferents and TNC [58].

In summary, the role of TRP channels in migraine mechanosensitivity presents a complex landscape with potential benefits for targeted therapies, but challenges still remain inviting more studies of this heterogeneous family of channels with the specific profile of the activators and inhibitors.

### Mechanosensitive K2P channels implicated in anti-nociception

In this systematic review, 9 articles discussed K2P channels involvement of mechanosensitive mechanisms in migraine [37, 84–90]. Two representatives of K2P channels, TREK1 and TREK2 are expressed in nociceptive small and medium fibers and their activity is triggered by mechanical stimuli such as stretch, but also by temperature, low pH and the non-steroidal anti-inflammatory drug BL-1249 [41, 91]. Moreover, it has been shown that the three types of TREK channels can co-assemble not only with each other, but also with other K2P channel members, assuming different functions [91]. Thus, it was observed that wild type TRESK and TREK2 subunits co-assemble forming a common functional heterodimers in TG neurons [37].

TRESK is also one of such partners for other K2P channels and the only K2P channel regulated by intracellular calcium concentration through calcineurin-mediated phosphorylation [89, 92]. TRESK channels in trigeminal neurons can be activated by cell swelling and inhibited by cell shrinkage [86]. Indeed, while negative pressure causes a 1.51-fold increase in channel opening probability, arachidonic acid, acidic pH and hypertonic stimulation, stimulating cell shrinkage, prevent TRESK opening, which is typically observed in inflammatory states [86]. In line with this, it has been proposed that several key mediators released during inflammation could modulate sensory transduction through small changes in membrane tension. Lengyel et al. showed the potential of the anti-amoebic drug cloxyquin effectively activate TRESK channels with a pronounced effect on channels in the resting state [84]. Similarly effective were several chemically modified analogs of cloxyquin [89].

In a recent study, a frameshift mutation responsible for the expression of non-functional TRESK subunits, has been discovered in a family suffering from migraine with aura [38]. One of these non-functional subunits produces a second protein fragment with a mechanism known as frameshift mutation-induced alternative translation initiation (fsATI) [38]. This second protein was shown to inhibit the action of TREK1 and TREK2 channels in trigeminal sensory neurons causing mechanical allodynia in migraine models [38, 85]. Furthermore, in the same study, by using double knockout mice for TREK1 and TREK2, TRESK mutant increased neuronal excitability by inhibiting TREK1 and TREK2 [38]. In contrast, activation of TREK channels inhibited the neuronal excitability and prevented release of pro-inflammatory peptides, thus suppressing migraine pain symptoms [85]. To further investigate the functions of the mutant TRESK subunit, Liu et al. showed via current-clamp recordings that neurons expressing mutant TRESK subunits have a lower threshold for action potential initiation and a higher spike frequency upon activation [90]. These findings propose that the mutation leads to an overexcitable state in trigeminal neurons and could potentially facilitate a migraine attack [90]. To further confirm TREK role in migraine origin, Kang et al. demonstrated their expression in rat medial vestibular nuclei, which may associate them to vestibular migraine symptoms, including vertigo or ataxia [88].

Instead, the active compound of Sichuan pepper, sanshool, has been shown to inhibit K2P channels TRESK, TASK1 and TASK3 [93]. Sanshool increases the frequency of action potentials and stimulates a specific burst pattern in mechanosensitive subpopulation of sensory neurons in the skin [93]. As a result, tingling paraesthesia occurs particularly via TRESK channel inhibition [87]. TRESK channels activity has been also been observed in DRG neurons stimulated by radial stretch [92]. The over-expression of TRESK in TG neurons increased potassium ion currents and decreased in the excitability of small-diameter TG neurons [87]. Therefore, TRESK-specific channel openers may exhibit analgesic effect by reducing the excitability of trigeminal primary afferent neurons [87].

In summary, K2P channels' role in mechanosensitive mechanisms of migraine, highlights the functional significance of TREK1 and TREK2 expression in sensory neurons, while the discovery of a frameshift mutation in TRESK subunits is linking them to migraine pain modulation. Additionally, a unique, among other K2P channels regulation of TRESK by intracellular calcium and sensitivity to cloxyquin, makes this channel a potential target for analgesia in migraine.

### The role of putative mechanosensitive Piezo channels in migraine

Piezo 1 and Piezo2 are two subtypes of recently discovered [42] highly mechanosensitive ion channels. Their expression in sensory neurons [42, 94] suggests them as the first candidates for detection of even tiny mechanical forces which might result in pain signalling in migraine. However, to date, only five papers were selected by the string on the involvement of Piezo channels in migraine nociception. In particular, Piezo1 was confirmed to be functionally expressed not only in the TG neurons [94], but also in trigeminal satellite glial cells [95], as well as in meningeal afferents [76, 94], where migraine pain originates from. Indeed, in ex vivo hemiskull meningeal preparation, the specific Piezo1 agonist Yoda1 activated sustained nociceptive spiking activity in the trigeminal nerve fibers [94]. Further, in the other paper, Piezo1 role has been shown in vivo, resulting in activation of brainstem neurons after dural application of low doses of the specific Piezo1 agonist Yoda1, while the same agonist reduced neuronal activity at higher dose [96].

Even though Piezo1 has been proposed as a target to develop analgesic effect in migraine, till recently, there was still a lack of a comprehensive knowledge on the role of this channel in different types of cells constituting trigeminovascular system. In this regard, two recent papers extended the knowledge of Piezo1 role in migraine nociception. Thus, one study found a remarkable property of the fluorescent dye FM1-43 to track previous nociceptive activity associated with activation of Piezo1 channels in the trigeminal nociceptive system [95]. In the other study, Piezo1 activity was compared in trigeminal *versus* DRG neurons using a microfluidic chip with reduced shear stress conditions [97]. This research surprisingly uncovered a higher activity of membrane located Piezo1 channels in DRGs compared to trigeminal cells while the level of mRNA was higher in trigeminal neurons suggesting the latent ability of Piezo1 channels to be upregulated upon sensitization in migraine conditions by engaging he intracellular pool of these channels.

Piezo2 subtype, as Piezo1, is also expressed in DRG and TG neurons [94, 98]. However, the lack of a specific agonist for Piezo2 makes it difficult to investigate its functional activity in migraine mechanosensitivity.

To sum up, although Piezo channels are certainly implicated in pain signalling and functionally expressed in the trigeminal nociceptive system, further research is needed to consider whether these recently discovered mechanotransducers can serve as appropriate targets for analgesic treatments in migraine.

### Acid sensitive ion channels blockage as targeted migraine medication

ASICs primarily sensitive to acid environment, have been identified as potential targets for new migraine medications to modulate mechanical pain in only 3 papers. Systemic injections of amiloride and mambalgin-1 reversed acute cutaneous mechanical allodynia in rats by inhibiting channels containing ASIC1a and ASIC1b subunits [99]. Another study has shown that ASIC3 subtype plays a crucial role in low pH evoked dural afferent activation and migraine-related pain behaviour [100]. Notably, the periorbital mechanosensitivity induced in mice by NTG and bright light stress-evoked latent sensitivity are reversed by the ASIC3 blocker APETx2 [101] suggesting their link to migraine-related mechanotransduction.

These studies reveal promising potential for treating migraine by targeting ASICs with specific inhibitors like amiloride, mambalgin-1, and APETx2. They also emphasize the specific role of the ASIC3 subtype in migraine-related mechanosensitive pain behavior.

### NMDA receptors as potential mechanotransducers in migraine pain

Mechanosensitivity of NMDA receptors (an alternative opening to conventional glutamate induced activation) attracted growing interest recently [47, 102]. In our search, among the two studies connecting NMDA receptors to migraine mechanical symptoms, one paper suggests that sec-butylpropylacetamide (SPD), a valproic acid derivative currently in use for migraine prophylaxis, is responsible for enhancing GABAergic transmission while reducing NMDA-mediated currents in cortical neurons [103]. These findings highlight SPD's potential as a promising anti-migraine compound reducing excessive neuronal excitability. In the other paper, in a NTG-induced mouse model of chronic migraine, while the beta blocker propranolol effectively reduced NTG-induced hyperalgesia, the valproic acid and NMDA receptor antagonist memantine showed a limited anti-nociceptive efficacy [104]. Although these studies present promising insights, further research is needed to fully understand the molecular mechanism of NMDA channels opening by mechanical forces and explore a perspective and potential mechanisms of NMDA blockers in reduction of mechanical pain at peripheral and central sites.

### Sex-differences in mechanosensitivity of migraine pain

Migraine is about three to four times more prevalent in women than in men [30]. Furthermore, women tend to experience more severe and disabling migraine, this sex disparity is probably linked to hormonal differences, as evidenced by increased migraine rates post-menarche, reaching a peak in their thirties, and a steep decline after menopause. While the exact mechanisms of these events in migraine patients remain unclear, animal studies have recently started to underscore sex differences in mechanosensitivity mechanisms of migraine [76]. Thus, new data have emerged on the modulation and activation of various ion channels involved in pain transmission by sex hormones. Indeed, our search identified sex differences associated with mechanosensitive receptors in migraine pain in two articles. One of these studies suggested that mechano- and sex hormones-sensitive TRPM3 channels may play a significant role in the prevalence of migraine in females. Female mice exhibited much higher, than males, nociceptive responses of meningeal afferents to two different TRPM3 agonists including the endogenous compound PregS (Table 2). In addition, females showed a twofold increase in the number of “super-mechanosensitive” nociceptive fibers co-expressing mechanosensitive TRPM3 and Piezo1 channels [76].

In the other paper, Cohen et al. (2021) investigated in preclinical NTG migraine model, the role of the mechanosensitive TRPC4 channels in trigeminal pain by using the TRPC4 antagonist ML204 (Table 2). Notably, both males and female mice responded similarly to the antinociceptive action of ML204 by reduced mechanical hypersensitivity, linked to decreased level of the migraine related neuropeptide CGRP [63]. This fits with lack of sex dependence of Piezo1 and TRPV1 mediated signalling in meningeal afferents [76].

Despite these emerging novel data on the sex-based differences in receptor mechanisms underlying mechanosensitivity in migraine, the full extent of this presumably complex mechanisms remains incompletely understood, requiring additional research in this area.

Our systematic review highlights a significant research gap in the field of migraine mechanoreceptors, particularly regarding the core topic of sex differences in migraine.

### Limitations

A significant general limitation of the research on mechanosensitive receptors in the context of migraine, is the historical separation of pain and migraine studies. Migraine headache and other types of pain have traditionally been investigated as distinct domains, often with different research communities and with distinct models and methodologies. It is not surprising given that the clinical manifestations of migraine pain are mainly distinctive from pain induced by nerve damage, cancer or inflammation. Important difference, for instance, is that migraine presents a specific risk profile related to medication overuse, emphasizing the need for tailored treatment approaches. Nevertheless, this separation has limited the extent to which mechanosensitivity, a common factor in both pain and migraine, has been explored comprehensively enriching both domains.

Integrating these two areas of study is a relatively recent endeavour, and as a result, the understanding of how mechanosensitive receptors intersect with migraine remains incomplete. It is noteworthy that this review was formally limited to the relationship between migraine and mechanosensitivity, not including pain itself. Although the findings on mechanosensitivity of other types of pain could help to better understand migraine pain, we did not include them in the main results of this review.

Moreover, we were also prevented from the theme of the review to present novel data coming from basic molecular mechanisms of mechanotransduction in studies unrelated to migraine. For instance, several recent studies identified a new class of high-threshold non-selective cationic channels transmembrane 63 (TMEM63), present in mammals that might act in parallel with Piezo1 [105]. Therefore, TMEM63 as well as the structurally similar mechanosensitive TMC1/2 [106] could also be potential players as mechanosensitive triggers of migraine nociception. However, their potential role in nociception deserves further investigations.

Furthermore, it is crucial to consider that mechanosensitivity property could extend beyond ionotropic receptors, being even more common in other receptor types, including metabotropic receptors. This aspect represents a further limitation that merits future exploration.

The present review focuses on potential mechanisms of peripheral sensitization in migraine, as it is mechanistically clear how mechanosensitive receptors such as calcium permeable Piezo1 or TRP channels may, on the one hand, facilitate the release of CGRP, and on the other hand, serve as transducers of mechanical forces into nociceptive electrical firing. However, although migraine is also characterized by central sensitization, analysis of the contribution of mechanosensitive receptors to central phenomena such as allodynia is limited by the still uncertain site of origin and unclear mechanisms of this migraine symptom. Better understanding of allodynia may have an important therapeutic impact as has been recently described by Ashina et al. (2023), who observed that non-ictal cephalic allodynia can be used to identify galcanezumab responders and non-responders.

Other related studies have focused on mechanical sensitivity throughout the different migraine phases. To this end, Scholten-Peeters et al. (2020) showed that people with migraine have enhanced mechanical sensitivity in cephalic and bilateral extra-cephalic regions, at the dominant and non-dominant side of migraine, compared to healthy participants. This enhanced mechanical sensitivity was more notable in periods just before (preictal), during (ictal), and after (postictal) a migraine attack, with the most significant reduction in PPT during the ictal phase.

However, a limitation of the current literature is that clinically observed changes in mechanosensitivity are not associated with the identification of specific molecular mechanisms involving mechanosensitive receptors. Thus, this limitation highlights the emerging need to accelerate the transition from preclinical studies of mechanotransduction in animal models to new assays measuring activity of mechanosensitive receptors in migraine patients.

Important also to note that Piezo and K2P channels are putative mechanosensitive channels, meaning that they are primarily mechanically gated channels that act as the specific force sensors themselves [107–109], while TRP, ASICs and NMDA channels are likely primarily designed to react to other specific stimuli making their mechanical sensitivity a “second profession”. As for glutamate NMDA receptors, in this review, we limited discussion on the role of NMDA channels to their pure mechanosensitive role while they play a key contribution to CSD underlying migraine aura [110] and participate in transmission of nociceptive stimuli. Worth noting that sensitivity to various stimuli can bring to polymodal receptors a property to serve as coincidence detectors of mechanical forces and chemical signals, a phenomenon that we have not discussed here.

In summary to this part, further research is needed to address these limitations and gain a more comprehensive understanding of the mechanosensitive mechanisms underlying migraine. Despite these limitations, this systematic review sheds light on the potential involvement of several subtype of mechanosensitive receptors in migraine, and we believe, it represents a further step towards understanding this complex migraine pathology, in particular, migraine with dominating symptoms of mechanical pain. Mechanosensitivity in migraine is likely to exhibit variability across patients [111], which can make it is not easy to develop universally effective treatments. Therefore, understanding the role of mechanosensitive receptors in individual cases and tailoring treatments accordingly, presents a considerable challenge for researchers and clinicians.

## Conclusions

In summary, our analysis indicates that, despite growing global interest to the biological role of mechanosensitive receptors and apparent progress in the emerging field of *Mechanoneurobiology*, the functions of these nociceptive transducers in migraine pathology have received a limited attention.

**Fig. 4:**
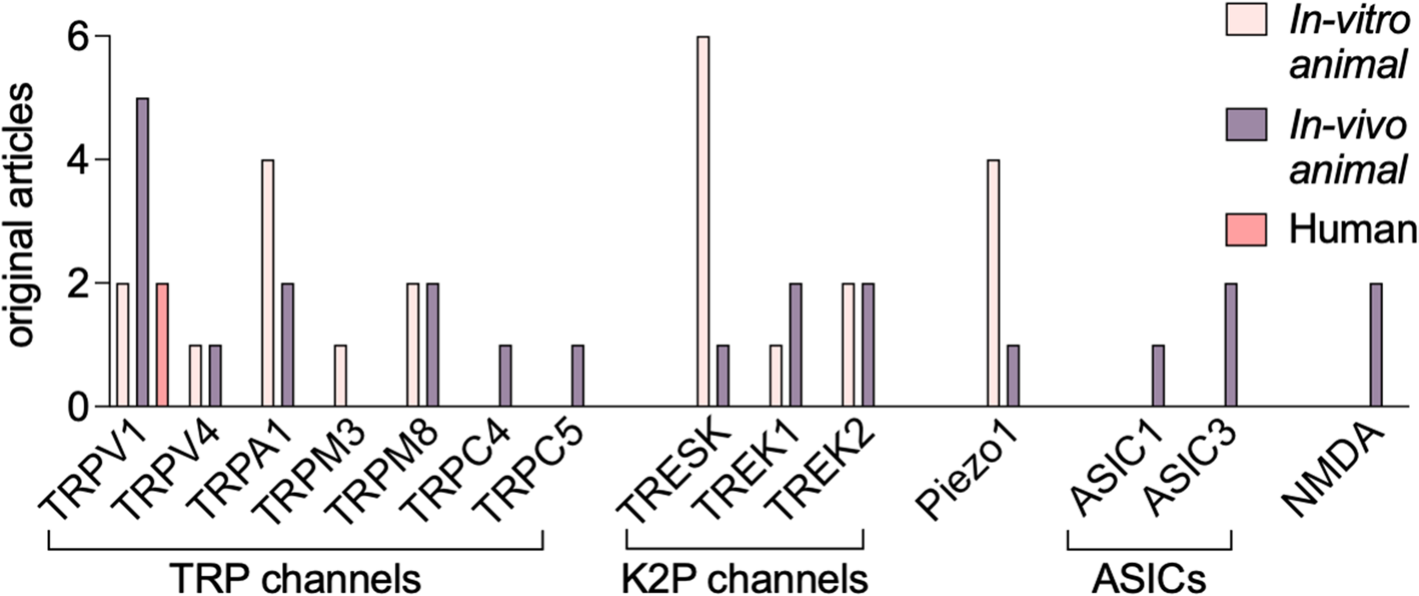
Mechanosensitive channels in migraine pain and their research stages. As evidenced from our searching results, polymodal, including sensitivity to mechanical forces, TRP channels are the most studied in migraine. The studies have been focused on TRPV1, TRPV4, TRPA1, TRPM3, TRPM8, TRPC4, TRPC5 subtypes with the best evidence for TRPV1 channels. For these channels, evidence comes from *in-vitro* and *in-vivo* studies, with one study on human tissues but includes one failed clinical trial on TRPV1 receptors as the target for migraine medication. For K2P and Piezo channels, their mechanosensitivity in migraine has been proposed for TRESK, TREK1, TREK2, and Piezo1 in both *in-vitro* and *in-vivo* animal studies. Instead, ASIC channels, including ASIC1 and ASIC3, and NMDA receptors are at the *in-vivo* animal migraine models research stage

Figure 4 provides a summary of current evidence, obtained from in vitro, in vivo animal and human studies, on involvement of certain type of mechanosensitive receptors in migraine pathology. This summary indicates that the most underdeveloped area is the testing the role of mechanosensitive receptors in human cells and tissues and related translational aspects of mechanobiology.

Among the mechanotransducers studied, members of the TRP family are most widely represented in the nociceptive system. The most studied TRPV1 channels, although present in the trigeminal nociceptive system and upraised during neuronal sensitization, still raise questions about their suitability as drug targets given the side effects of TRPV1 antagonists, which were failed in clinical trials [112]. Other TRP channels, such as TRPV4, TRPM3, TRPC4 and TRPM8, remain reliable candidates to be involved in migraine pain signaling with potential to be drug targets. The mechanosensitive K2P channels TREK1 and TREK2, in co-assembly with K2P TRESK subunits with frameshift mutations implicated in migraine, may also be considered for novel pharmacological interventions acting via unconventional antinociceptive mechanism. Piezo channels are of particular interest in the mechanobiology of migraine given their hypersensitivity to tiny mechanical forces and high calcium permeability, but further research is needed to understand their role in migraine. The latter may be facilitated by the development of new pharmacological tools to block the function of these newly discovered channels, with a focus not only on Piezo1 but also on the little studied in migraine Piezo2 subtype. ASICs, including ASIC and ASIC3, have potential as targets for the treatment of migraine, and inhibitors such as amiloride, mambalgin-1, and APETx2 that showed a promising alleviating mechanical allodynia effect in animal models. NMDA receptors are clearly involved in migraine central and likely, in peripheral nociceptive mechanisms. However, their translational impact is limited given their critical role in essential brain functions. Although targeting mechanosensitive channels in migraine therapy has significant potential for preventing mechanical pain, the development of effective treatments certainly requires more research.

## Data Availability

No datasets were generated or analysed during the current study.

## Abbreviations

ASICs: Acid-sensing ion channels
ATP: Adenosine triphosphate
CFA: Complete freund’s adjuvant
CGRP: Calcitonin gene related peptide
CNS: Central nervous system
CSD: Cortical spreading depression
K2P: Two-pore-domain potassium channel
LPC: Lysophosphatidylcholine
MO: Mustard oil
NMDA: N-methyl-D-aspartate receptors
NTG: Nitroglycerin
PregS: Pregnenolone sulfate
TALK: TWIK-related alkaline pH-activated K + channel
TASK: TWIK-related acid-sensitive K + channel
TG: Trigeminal ganglia
TMC1/2: Transmembrane channel-like 1/2
TMEM63: Transmembrane 63
TNC: Trigeminal nucleus caudalis
TRP: Transient receptor potential
TRPA: Transient receptor potential ankyrin
TRPC: Transient receptor potential canonical
TRPM: Transient receptor potential melastatin
TRPV: Transient receptor potential vanilloid
TREK: TWIK-related K + channel
TRSK: TWIK-related spinal cord K + channel
TWIK: Weak rectifying K + channel
UMO: Umbellulone
